# A GH115 α-glucuronidase structure reveals dimerization-mediated substrate binding and a proton wire potentially important for catalysis

**DOI:** 10.1107/S2059798322003527

**Published:** 2022-04-20

**Authors:** Casper Wilkens, Marlene Vuillemin, Bo Pilgaard, Igor Polikarpov, Jens Preben Morth

**Affiliations:** a Department of Biotechnology and Biomedicine, Technical University of Denmark, Søltofts Plads 224, 2800 Kongens Lyngby, Denmark; b São Carlos Institute of Physics, University of São Paulo, Avenida Trabalhador Sãocarlense 400, 13566-590 São Carlos, SP, Brazil

**Keywords:** α-glucuronidases, dimerization, glycoside hydrolase family 115, metal dependence, Grotthuss mechanism

## Abstract

Xylan is a major constituent of plant cell walls and is a potential source of biomaterials, and the derived oligosaccharides have been shown to have prebiotic effects. Xylans can be highly substituted with different sugar moieties, which pose steric hindrance to the xylanases that catalyse the hydrolysis of the xylan backbone. One such substituent is α-d-glucuronic acid, which is linked to the O2′ position of the β-1,4 d-xylopyranoses composing the main chain of xylans. The xylan-specific α-glucuronidases from glycoside hydrolase family 115 (GH115) specifically catalyse the removal of α-d-glucuronic acid (GlcA) or methylated GlcA (MeGlcA). Here, the molecular basis by which the bacterial GH115 member wtsAgu115A interacts with the main chain of xylan and the indirect involvement of divalent ions in the formation of the Michaelis–Menten complex are described. A crystal structure at 2.65 Å resolution of wtsAgu115A originating from a metagenome from an anaerobic digester fed with wastewater treatment sludge was determined in complex with xylohexaose, and Asp303 was identified as the likely general acid. The residue acting as the general base could not be identified. However, a proton wire connecting the active site to the metal site was observed and hence a previous hypothesis suggesting a Grotthuss-like mechanism cannot be rejected. Only a single molecule was found in the asymmetric unit. However, wtsAgu115A forms a dimer with a symmetry-related molecule in the crystal lattice. The xylohexaose moieties of the xylohexaose are recognized by residues from both protomers, thus creating a xylohexaose recognition site at the dimer interface. The dimer was confirmed by analytical size-exclusion chromatography in solution. Kinetic analysis with aldouronic acids resulted in a Hill coefficient of greater than 2, suggesting cooperativity between the two binding sites. Three Ca^2+^ ions were identified in the wtsAgu115A structures. One Ca^2+^ ion is of particular interest as it is coordinated by the residues of the loops that also interact with the substrate. Activity studies showed that the presence of Mg^2+^ or Mn^2+^ resulted in a higher activity towards aldouronic acids, while the less restrictive coordination geometry of Ca^2+^ resulted in a decrease in activity.

## Abstract 2

The crystal structure of a GH115 α-glucuronidase obtained in complex with xylohexaose and Ca^2+^ reveals that the two molecules constituting the homodimer cooperatively bind the substrate and that a divalent ion is involved in formation of the Michaelis–Menten complex and is likely to contribute to the formation of a protein wire that is essential for catalysis.

## 1. Introduction

Xylans are the main hemicellulosic constituents found in the lignified secondary cell walls of trees in particular and can account for up to a third of the dry weight of the plant cell wall (Scheller & Ulvskov, 2010 ▸). Enzymes that degrade and modify xylan polysaccharides are considered to be attractive for use in biorefining industries, where xylans have shown promise as biomaterials, for example in hydrogels for wound dressing and drug delivery and in packaging materials (Naidu *et al.*, 2018 ▸), and the derived oligosaccharides have been shown to have prebiotic effects (Santibáñez *et al.*, 2021 ▸). A significant xylan component is glucuronic acid, a substituent that is found on many different types of xylan, where the O1 of α-d-glucuronic acid (GlcA) is linked to the O2′ position of the β-1,4-linked d-xylopyranose constituting the main chain of the xylan. The GlcA unit of xylan often carries methyl substitutions at the 4-*O* position (MeGlcA), which are believed to form inter­action points for lignin and cellulose (Peña *et al.*, 2016 ▸; Bromley *et al.*, 2013 ▸). Xylan-specific α-glucuronidases (EC 3.2.1.131) belong to glycoside hydrolase family 115 (GH115) in the Carbohydrate Active enZyme database (CAZy; http://www.cazy.org; Drula *et al.*, 2021 ▸). Xylan-specific α-gluc­uronidases specifically act on the α-d-glucuronic acid (GlcA or MeGlcA) substituents present in polymeric and oligomeric xylan (Ryabova *et al.*, 2009 ▸; Rogowski *et al.*, 2014 ▸; Chong *et al.*, 2011 ▸), while other accessory enzymes of the GH115 family act on different substituents such as ferulic acid, arabinose and acetyl groups (Yan *et al.*, 2021 ▸; Holck, Freds­lund *et al.*, 2019 ▸; Kmezik *et al.*, 2020 ▸). These substituents make xylans recalcitrant to enzymatic degradation (Lyczakowski *et al.*, 2017 ▸; Kmezik *et al.*, 2020 ▸; Couturier *et al.*, 2018 ▸) and hence should be hydrolysed before β-1,4-xylanases (EC 3.2.1.8) and β-1,4-xylosidases (EC 3.2.1.3) can completely degrade the xylan (Holck, Djajadi *et al.*, 2019 ▸; Kmezik *et al.*, 2020 ▸). Thus, GlcA or MeGlcA substituents present a bottleneck in the degradation of xylan, and as such are crucial targets for bio­refineries relying on the enzymatic degradation and utilization of unsubstituted xylan in biomaterials.

Four crystal structures of GH115 α-glucuronidases have been determined to date (Yan *et al.*, 2021 ▸; Rogowski *et al.*, 2014 ▸; Aalbers *et al.*, 2015 ▸; Wang *et al.*, 2016 ▸). However, only the structure of the GH115 member from *Bacteroides ovatus* (*Bo*Agu115A) was obtained in a complex with a ligand, d-guluronic acid. In combination with comprehensive mutational analysis, the specific location of d-guluronic acid was identified to be the active site (Rogowski *et al.*, 2014 ▸; Wang *et al.*, 2016 ▸). The crystal structures of *Bo*Agu115A and other GH115 orthologs from *Saccharophagus degradans* (*Sde*Agu115A) and *Amphibacillus xylanus* (*Axy*Agu115A) are considered to be active dimers (Yan *et al.*, 2021 ▸; Rogowski *et al.*, 2014 ▸; Wang *et al.*, 2016 ▸). In *Bo*Agu115A, a structural and mutational study identified the residues at the dimer interface that are involved in binding the xylan (Rogowski *et al.*, 2014 ▸). Only the GH115 ortholog from *Bacteroides thetaiotaomicron* (*Bt*GH115A) is believed to function as a monomer (Aalbers *et al.*, 2015 ▸). The importance of dimerization in the catalytic mechanism remains elusive, in part due to a lack of structural information on complexes with xylo-oligosaccharides that mimic the xylan backbone.

*Bt*GH115A is the only characterized GH115 member that shows activity on arabinogalactan, from which it can release α-GlcA, according to Aalbers *et al.* (2015 ▸); however, GlcA is linked to the galactose in arabinogalactan *via* a β-linkage (Tryfona *et al.*, 2012 ▸). It does not show activity on GlcA or MeGlcA linked to xylan (Aalbers *et al.*, 2015 ▸). Should *Bt*GH115A be active on a β-linkage this would be highly unusual, as families in CAZy typically display activity on either α-linkages or β-linkages (The CAZypedia Consortium, 2018 ▸).

Several metal ions have been observed in the crystal structures of GH115 members, Na^+^ in the crystal structure of BoAgu115A, Ni^2+^ in that of *Bt*GH115A and Na^+^ in that of *Sde*Agu115A (Rogowski *et al.*, 2014 ▸; Aalbers *et al.*, 2015 ▸; Wang *et al.*, 2016 ▸), but these have received little or no attention as their potential role in the catalytic mechanism has not previously been investigated.

In this study, we report crystal structures of a bacterial GH115 ortholog (wtsAgu115A) identified in a metagenome from an anaerobic digester fed with wastewater treatment sludge (Wilkens *et al.*, 2017 ▸). Crystal structures with and without bound xylohexaose are presented. The xylohexaose-bound crystal structure forms a Michaelis–Menten-like complex, which reveals how the dimer interface of the enzyme orients xylan in the active site guided by residues from each protomer of the homodimer. Further, wtsAgu115A displayed increased activity in the presence of divalent metal ions. In the crystal structure, a Ca^2+^ ion was identified in proximity to the active site, which coordinates some of the residues interacting with the xylohexaose and is also part of a proton wire passing through the active-site cavity. While Ca^2+^ resulted in a slightly decreased activity, the addition of Mg^2+^ and Mn^2+^ to the enzymatic assays resulted in increased activity, indicating the advantages of a more rigid coordination geometry around the divalent metal site for optimal activity. We hypothesize that the proton wire potentially delivers the proton needed for catalysis.

## 2. Materials and methods

### 2.1. Sequence analysis

The gene encoding wtsGH115A (GenBank Accession No. BK059344) was identified on contig MTKX01000768.1, base pairs 45336–47131 in a metagenomic study of anaerobic digesters Randers (Whole Genome Shotgun Accession No. MTKX00000000) fed with surplus sludge from municipal wastewater treatment, where it was predicted to originate from the bacterial genus *Sulfurovum* (Wilkens *et al.*, 2017 ▸). A 19-amino-acid signal peptide was identified by *SignalP* 4 (Petersen *et al.*, 2011 ▸). The molecular mass and pI were predicted by *Compute pI* (Gasteiger *et al.*, 2005 ▸): the pI was 4.7 and the molecular mass was 97.1 kDa. The theoretical molar extinction coefficient, calculated using *ProtParam* (Gasteiger *et al.*, 2005 ▸), was 184 040 *M* ^−1^ cm^−1^.

### 2.2. Multiple alignment and phylogenetic analysis

The identity of wtsGH115A to other GH115 members was calculated with *Clustal Omega* (Li *et al.*, 2015 ▸), which was also used for the multiple sequence alignment of all GH115 members listed in CAZy from which fragments were removed. The multiple alignment was used to build the LGmaximum likelihood phylogenetic tree using *RaxML-HPC BlackBox* (version 8.2.10; Stamatakis *et al.*, 2008 ▸) at the CIPRES Science Gateway version 3.3 (Miller *et al.*, 2010 ▸). *RAxML* stopped the rapid bootstrap search after 250 replicates with the MRE-based bootstopping criterion.

### 2.3. Cloning, expression and purification

A codon-optimized mature gene (Supplementary Fig. S1) for *Escherichia coli* encoding wtsAgu115A was purchased and cloned into pET-28a using the NcoI and XhoI restriction sites (GenScript). The resulting plasmid was transformed into *E. coli* strain BL21 (DE3) pLysS (Novagen). Transformants were grown in LB medium (2 × 500 ml, 37°C, 18 h), harvested at 2000*g* for 10 min at room temperature (RT), resuspended in 100 ml M9 medium (see below) and used to inoculate M9 medium (4 l) supplemented with 1%(*w*/*v*) glucose, 2 ml l^−1^ trace elements (Holme *et al.*, 1970 ▸) and 50 µg ml^−1^ kanamycin in a 5 l bioreactor (Sartorius Biostat Aplus). Glucose [50%(*w*/*v*)] supplemented with 10 ml l^−1^ trace elements (Holme *et al.*, 1970 ▸) was used as a carbon source during the fed-batch phase. The culture was propagated at 37°C until the OD_600_ reached 12, whereupon the temperature was decreased to 15°C and expression was induced by the addition of isopropyl β-d-1-thiogalactopyranoside (IPTG) to a final concentration of 1 m*M*. The pH was maintained at 7 by adding 28%(*w*/*v*) ammonia; the temperature and dissolved oxygen tension were controlled automatically. After 16–18 h, the cells were harvested (2000*g*, 20 min, 4°C) and stored at −20°C. The cells were resuspended in buffer *A* (50 m*M* Tris–HCl, 0.5 *M* NaCl, 20 m*M* imidazole pH 7.5) using 10 ml per gram of wet weight cells, lysed (Pressure Cell Homogeniser; Stansted Fluid Power) at RT, treated with benzonase nuclease (Invitrogen) for 30 min at RT and centrifuged (20 000*g*, 30 min, 4°C). The supernatant was filtrated (0.45 µm Durapore membrane filters; Millipore), applied onto a 5 ml HisTrap HP column (GE Healthcare) at a flow rate of 3 ml min^−1^ and eluted with a linear imidazole gradient (0.02–0.5 *M*) in buffer *A*. Fractions containing wtsGH115A were pooled, dialysed [SnakeSkin (3.5 kDa), Thermo Scientific] into 10 m*M* sodium acetate pH 6 and applied onto a 6 ml Resource Q column (GE Healthcare) equilibrated with 10 m*M* sodium acetate pH 6 at a flow rate of 3 ml min^−1^ and eluted with a linear 0–0.5 *M* NaCl gradient (30 column volumes). The fractions containing wtsGH115A were pooled, concentrated [Vivaspin (10 kDa), Sartorius] and then applied at a flow rate of 0.5 ml min^−1^ onto a HiLoad 16/60 Superdex G200 column (GE Healthcare) equilibrated with 10 m*M* sodium acetate, 150 m*M* NaCl pH 6. The fractions containing wtsAgu115A were concentrated to 139 µ*M* [Vivaspin (10 kDa), Sartorius] and stored at 4°C. All chromatographic steps were carried out at RT. The purity was checked on 12% SDS–PAGE gels (Supplementary Fig. S2). The concentration of the protein samples was measured as the absorption at 280 nm (*A*_280_) using an extinction coefficient of 184 040 *M* ^−1^ cm^−1^.

An inactive mutant, D303A, was constructed using CloneAmp polymerase (Takara, Kusatsu, Japan), a set of mutagenic primers (Supplementary Table S1) and pET-28a/GH115 WT as a template. The template was digested by DpnI at 37°C overnight and the PCR products were purified using the Illustra GFX PCR DNA and Gel Band Purification Kit (GE Healthcare Life Sciences, Chicago, Illinois, USA). Purified pET-28a/GH115 D303A was transformed into *E. coli* DH5α for replication and plated on LB plates supplemented with kanamycin (50 µg ml^−1^). Positive transformants were selected and the corresponding plasmids were extracted using a GeneJET Plasmid Miniprep Kit (ThermoFisher Scientific). All constructs were checked by sequencing (Macrogen Europe, Amsterdam, Netherlands). The D303A mutant was expressed and purified as described for the wild type.

### 2.4. Analytical size-exclusion chromatography

Analytical gel filtration was performed on a HiLoad 16/60 Superdex G200 column equilibrated with aldolase (158 kDa), conalbumin (75 kDa) and ovalbumin (44 kDa) (all from GE Healthcare) dissolved in 10 m*M* sodium acetate, 150 m*M* NaCl pH 6 at a flow rate of 0.5 ml min^−1^ with 10 m*M* sodium acetate, 150 m*M* NaCl pH 6 as the running buffer.

### 2.5. Temperature optima and pH

A stock solution of an aldouronic acids mixture (Megazyme; 20 µl) was diluted to 1% in 40 m*M* Britton–Robinson universal buffers pH 2–10, 0.005% Triton X-100, mixed with 5 µl 24 µ*M* wtsGH115A and incubated for 10 min at 37°C to determine the pH optimum. Prior to mixing, the reaction mixture and wtsAgu115A were pre-incubated for 2 min at 37°C. The reaction was stopped by adding 25 µl 1 *M* trichloroacetic acid (TCA). According to the manufacturer’s recommendations, the release of GlcA was quantified by the d-Glucuronic/d-Galacturonic Acid Assay Kit (Megazyme). In brief, d-glucuronic acid is oxidized to d-glucarate by uronate dehydrogenase in the presence of NAD^+^, resulting in the formation of NADH, which can be detected at *A*_340_. The formation of NADH is stoichiometric with d-glucuronic acid. One activity unit (U) was defined as the amount of enzyme releasing 1 µmol GlcA per minute. The temperature optimum was determined at pH 6 in the range 20–60°C as above. All experiments were performed in triplicate.

### 2.6. Temperature stability and pH

wtsGH115A (20 µl at 24 µ*M*) was incubated at RT in 80 µl 40 m*M* Britton–Robinson universal buffers pH 2–10 for five days and the residual activity was quantified as described in Section 2.5. Similarly, the temperature stability was assessed by incubating 24 µ*M* wtsGH115A in 10 m*M* sodium acetate pH 6 at 37 and 50°C, respectively, and the residual activity was quantified as above.

### 2.7. Effect of divalent metal ions

The effect of divalent ions on enzyme activity was investigated by measuring the activity of the enzyme using 8.5 mg ml^−1^ aldouronic acids, 2 m*M* divalent ions (CaCl_2_, MgCl_2_, ZnCl_2_, MnCl_2_, NiCl_2_ or FeCl_2_) and 2.5 µ*M* GH115 at 37°C in 50 m*M* sodium acetate, 0.005% Triton X-100 pH 6. The reaction was stopped at regular time intervals by adding one volume of 1 *M* TCA. The release of GlcA was quantified as above. Prior to analysis, the enzyme was first incubated with 1 m*M* EDTA for 10 min to exclude the possibility of any metal being bound and EDTA was then removed by dialysis at 1:200(*v*:*v*) overnight twice.

The effect of MgCl_2_ on the enzyme activity was further investigated under standard assay conditions using MgCl_2_ concentrations varying from 0 to 10 m*M*.

### 2.8. Specific activity

For specific activity studies, the following substrates were prepared: 1% potato pectic fibre rhamnogalacturonan I, linear arabinan, potato galactan, soybean pectic fibre rhamno­galacturonan, beechwood xylan, sugar beet arabinan, rye arabinoxylan (high viscosity) and wheat arabinoxylan (in­soluble), aldouronic acids, tamarind xyloglucan (all from Megazyme), larch wood arabinogalactan, xanthan gum, birchwood xylan, oat spelt xylan, acacia tree gum arabic (all from Sigma) and corncob xylan (Carbosynth). The mixture was dissolved or suspended depending on the solubility of the compound in 20 µl 50 m*M* sodium acetate, 0.005% Triton X-100 pH 6 and was mixed with 5 µl 24 µ*M* GH115 to initiate the reaction. The reaction mixture was left to incubate for 10 min at 37°C. The reactions were stopped by adding 25 µl 1 *M* TCA. The release of GlcA was quantified as above.

### 2.9. Kinetic parameters

To determine the kinetic parameters of the enzyme, initial velocities were quantified as in Section 2.5 for aldouronic acid mixtures with concentrations varying from 0.5 to 6 mg ml^−1^ in 50 m*M* sodium acetate, 0.005% Triton X-100 pH 6 at 37°C using 2.5 µ*M* of the enzyme. The release of GlcA was quantified as described above. Kinetic parameters were determined by plotting initial velocities against initial substrate concentration and fitting the Hill equation, 

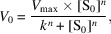

using the *Origin* 2019 software (OriginLab), where *V*_0_ is the initial velocity, [*S*_0_] is the initial substrate concentration, *V*_max_ is the maximum rate, *n* is the Hill coefficient and *k* is the half-maximal concentration constant.

### 2.10. Crystallization, data collection and data processing

The proteins wtsAgu115A and wtsAgu115A D303A (10–20 mg ml^−1^) in 10 m*M* sodium acetate, 150 m*M* NaCl pH 6 were crystallized in SuperClear plates (Jena Bioscience) by mixing 1 µl at 10 mg ml^−1^ with 1 µl reservoir solution consisting of 0.2 *M* CaCl_2_, 0.1 *M* HEPES pH 7.5, 28% PEG 400. Crystals containing xylohexaose (Megazyme) were obtained by adding a few xylohexaose crystals to the drops, which were then allowed to equilibrate overnight. The crystals were cryoprotected with 20% PEG 400 in reservoir solution for 5 s and cryocooled in liquid nitrogen. Data were collected on BioMAX at the MAX IV Laboratory (Ursby *et al.*, 2020 ▸) and on P14 at PETRA III. Data collection on BioMAX was performed using *MxCUBE*3 (Mueller *et al.*, 2017 ▸) and the data were processed and scaled with *xia*2 (Winter, 2010 ▸) to 2.30 Å resolution for the unbound protein and 2.65 Å resolution for the xylohexaose complex (Table 1 ▸). The xylohexaose geometry was validated using *Privateer* (Agirre *et al.*, 2015 ▸).

### 2.11. Phasing and refinement

The structure of unbound wtsAgu115A D303A was determined by molecular replacement with *Phaser* (McCoy *et al.*, 2007 ▸) from the *Phenix* package (Liebschner *et al.*, 2019 ▸) using *Bo*Agu115A (PDB entry 4c90) as the search model. This was the closest homolog from the PDB (https://www.wwpdb.org/) identified at the time through *PDB-BLAST* (Altschul *et al.*, 1997 ▸), with a coverage of ∼98% and an identity of ∼67%. The initial structure was built with *phenix.autobuild* (Terwilliger *et al.*, 2008 ▸) and then refined iteratively with *phenix.refine* (Afonine *et al.*, 2012 ▸) and manual model rebuilding in *Coot* (Emsley *et al.*, 2010 ▸) to a final *R*_work_ and *R*_free_ of 0.20 and 0.22, respectively. The wtsGH115A–xylohexaose structure (PDB entry 7pug) was determined using unbound wtsAgu115A D303A (PDB entry 7pxq) as the search model and was refined to a final *R*_work_ and *R*_free_ of 0.17 and 0.21, respectively.

### 2.12. Structural alignments

Structural alignments were obtained using *PyMOL* 2.3.3 (Schrödinger), which was also used to render structural models.

## 3. Results and discussion

### 3.1. Overall structure and comparison to other GH115 structures

**Figure 1:**
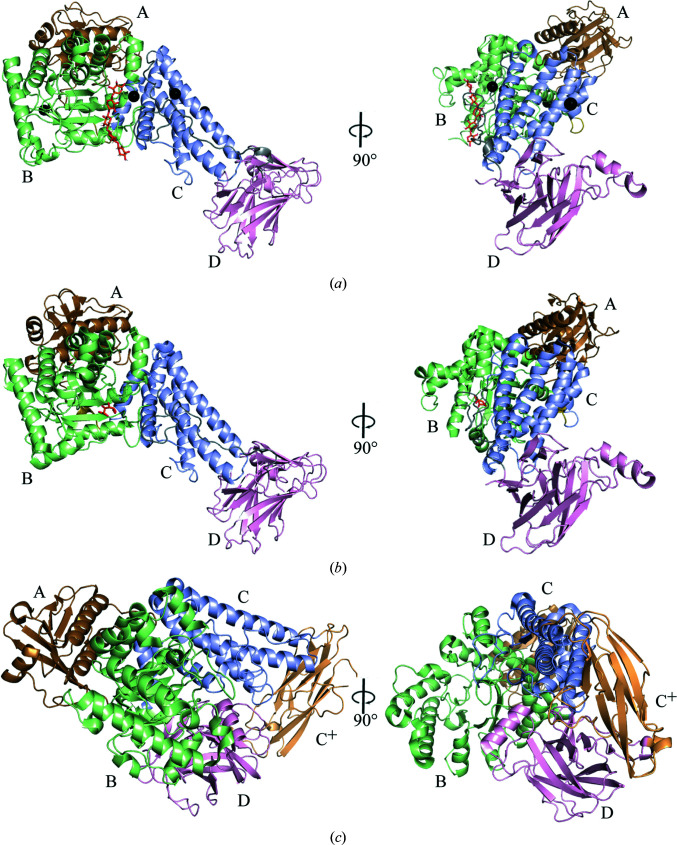
Overall structure of wtsAgu115A and other family members with the domains indicated. (*a*) wtsAgu115A in complex with xylohexaose (orange) and Ca^2+^ (black), (*b*) *Bo*Agu115A in complex with glucuronic acid (orange) (PDB entry 4c91) and (*c*) *Sde*Agu115A (PDB entry 4zmh).

The structure of the inactive mutant wtsAgu115A D303A in its unbound form was determined at 2.33 Å resolution (Table 1 ▸) with a single protomer in the asymmetric unit. The structures of wtsAgu115A and *Bo*Agu115A show an equivalent tertiary architecture (Figs. 1 ▸ *a* and 1 ▸ *b*) comprised of four consecutive domains, with the N-terminal domain A comprised of two perpendicular β-sheets and three α-helices (residues 1–169), domain B composed of a (β/α)_8_ TIM barrel (residues 170–468), domain C composed of an α-helix bundle (residues 474–617) and the C-terminal domain D composed of a canonical β-sandwich fold that includes two β-sheets of five antiparallel β-strands (residues 656–838) (Fig. 1 ▸ *a*). Domains B and C are connected by a five-residue loop and domains C and D are connected by a 38-residue loop (Fig. 1 ▸ *a*). The root-mean-square deviation (r.m.s.d.) for the C^α^ atomic coordinates of wtsAgu115A and *Bo*Agu115A was 0.49 Å (642 atom pairs). The GH115 members *Sde*Agu115A and *Axy*Agu115A have an additional domain, C^+^, which is not present in *Bo*Agu115A and wtsAgu115A, and also show a different domain organization (Fig. 1 ▸ *c*; Wang *et al.*, 2016 ▸; Yan *et al.*, 2021 ▸).

Both the unbound wtsAgu115A D303A structure and the wtsAgu115A–xylohexaose structure (2.65 Å) were refined to excellent crystallographic and geometric statistics, as summarized in Table 1 ▸. Three calcium ions were assigned to high electron-density sites found on the surface of domain B and domain C and connected to the active site in domain B (Fig. 1 ▸ *a*). Overall, the electron density is well defined in both unbound and xylohexaose-bound wtsAgu115A except for residues 301–318 and 307–317, respectively, suggesting that these residues constitute a flexible loop. The corresponding loops are also suggested to be flexible in *Bo*Agu115A, *Axy*Agu115A and *Sde*Agu115A and are believed to interact with the substrate (Wang *et al.*, 2016 ▸; Rogowski *et al.*, 2014 ▸; Yan *et al.*, 2021 ▸).

### 3.2. The Michaelis–Menten-like complex between wtsAgu115A and xylohexaose

**Figure 2:**
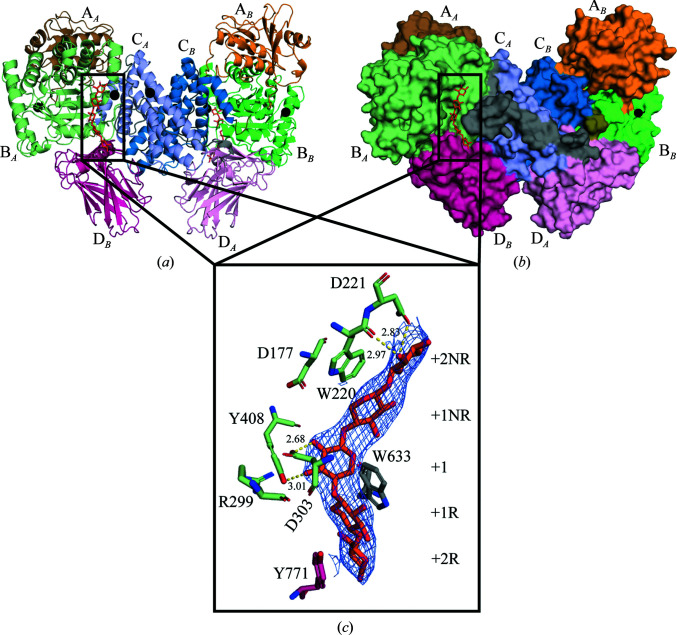
The Michaelis–Menten-like complex and wtsAgu115A dimerization, with domains indicated and chains indicated by subscripts. (*a*) Secondary-structure representation of the wtsAgu115A dimer in complex with xylopentaose (orange) and Ca^2+^ (black spheres), (*b*) surface representation of the wtsAgu115A dimer in complex with xylopentaose (orange) and Ca^2+^ (black spheres) and (*c*) enlargement of the five visible sugar moieties of the xylohexaose (orange) with hydrogen bonds shown (yellow dotted lines) with their distances in Å. The subsites are indicated and named according to McKee *et al.* (2012 ▸). d-Xylopyranose moiety 901 corresponds to +2NR, 902 to +1NR, 903 to +1, 904 to +1R and 905 to +2R.

**Figure 3:**
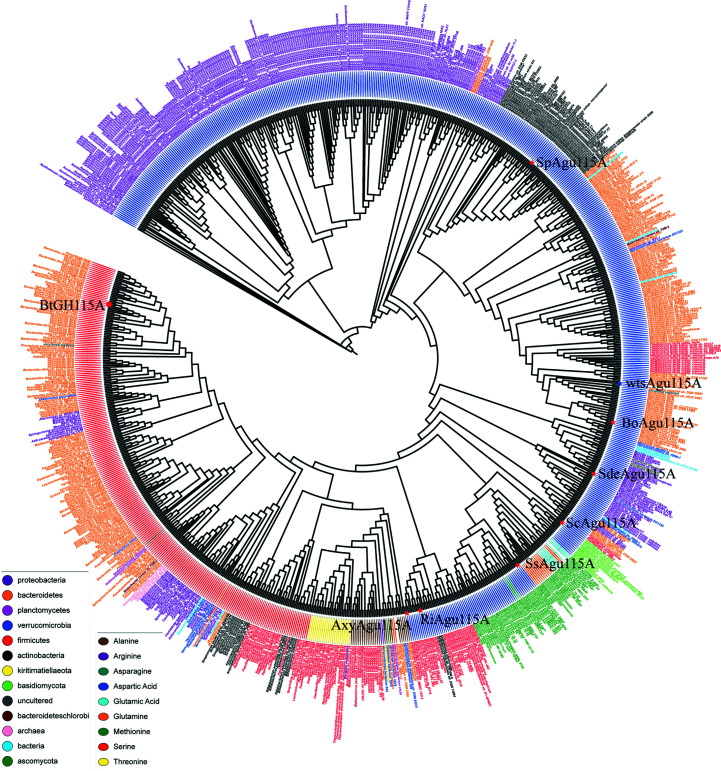
Phylogenetic tree for glycoside hydrolase family 115. The tree was visualized with *Interactive Tree of Life* (Letunic & Bork, 2021 ▸). The inner ring indicates the nonconserved residue potentially interacting with the metal ion present near the active site and the outer ring indicates the phyla. The characterized members are indicated by red dots and wtsAgu115A by a blue dot.

wtsAgu115A displayed the highest specific activity towards an aldouronic acids mixture, followed by beechwood and birchwood xylan, while no activity was observed on arabinogalactan, xanthan gum or gum arabic (Table 2 ▸), demonstrating that wtsAgu115A is a xylan-specific α-glucuronidase. Apparent and extended electron density for five β-1,4-linked d-xylopyranose moieties was observed in the crystal structure of wtsAgu115A soaked with xylohexaose, which was positioned in the central cavity of domain B (Figs. 2 ▸ *a* and 2 ▸ *b*). Superimposition with *Bo*Agu115A in complex with GlcA (PDB entry 4c91; Rogowski *et al.*, 2014 ▸) indicates that O2 of the +1 d-xylopyranose moiety would form the linkage to GlcA in the active-site pocket (Fig. 3 ▸), and thus this is subsite +1 according to McKee *et al.* (2012 ▸). The d-xylopyranose moieties adopt the stable ^4^ *C*_1_ chair conformation (Stortz, 2010 ▸). However, the xylohexaose deviates from the 3_1_-fold helical screw conformation observed for xylan in solution and the 2_1_-fold helical screw found on the surface of cellulose (Martínez-Abad *et al.*, 2017 ▸; Busse-Wicher *et al.*, 2014 ▸). The conformation of the xylan backbone is commonly expressed by two dihedral angles (φ and ψ) that refer to the rotations around the two C—O bonds that form the glycosidic linkages. The sum of φ (O5′—C1′—O4—C4) and ψ (C1′—O4—C4—C3) is indicative of the local conformation, and is ∼50° for a right-handed twisted 3_1_-fold helical screw conformation and ∼190° for the left-handed conformation (Busse-Wicher *et al.*, 2014 ▸). The sum of φ and ψ for the glycosidic linkages between the xylose moiety pairs at +2NR and +1NR, at +1NR and +1, at +1 and +1R and at +1R and +2R are 47°, 27°, 57° and 13°, respectively, which indicates that the xylan backbone is forced out of conformation upon forming the Michaelis–Menten complex. A structural comparison between unbound wtsAgu115A D303A and wtsAgu115A–xylohexaose yields an overall r.m.s.d. of 0.17 Å for C^α^ atoms, which suggests that the binding of xylohexaose does not impose any major domain conformational changes in wtsAgu115A.

A particular recognition site formed by the dimer is located at position +1NR; the d-xylopyranose moiety engages in CH–π stacking with Trp633 (Fig. 2 ▸ *c*). The tryptophan is fully conserved among structurally determined GH115 xylan-specific α-glucuronidases, but not among all GH115 members. However, this particular area of multiple sequence alignment is poor, making it difficult to decide which residues have replaced Tyr633 (see Supplementary File S1). In wtsAgu115A Trp633 ensures that the d-xylopyranose moiety at +1R is perpendicular to the active-site pocket (Fig. 2 ▸ *c*). The d-xylopyranose moiety at +2R forms an additional CH–π stacking with Tyr771 from the other protomer in the dimer (Figs. 2 ▸ *a*, 2 ▸ *b* and 2 ▸ *c*). In *Bo*Agu115A, an alanine mutant of the equivalent tyrosine resulted in a fivefold reduction in catalytic efficiency (Rogowski *et al.*, 2014 ▸), which demonstrates the importance of the dimerization observed for *Bo*Agu115A (Rogowski *et al.*, 2014 ▸) and also wtsAgu115A (Fig. 2 ▸ *c*). However, Tyr771 is not conserved in all GH115 xylan-specific α-glucuronidases (see Supplementary File S1), and structurally they deviate significantly from the equivalent in *Sde*Agu115A and *Axy*Agu115A that contain the C^+^ domain (Figs. 1 ▸ *a*–1 ▸ *c*). Tyr771 is conserved within the clades of the phylogenetic tree containing wtsAgu115A and *Bo*Agu115A (Fig. 3 ▸ and Supplementary File S1). Furthermore, wtsAgu115A eluted from a gel-filtration column at a volume consistent with a dimer in solution (Supplementary Fig. S3), which suggests that wtsAgu115A is a dimer in its active form similar to other GH115 xylan-specific α-glucuronidases (Wang *et al.*, 2016 ▸; Yan *et al.*, 2021 ▸; Rogowski *et al.*, 2014 ▸). The Hill coefficients of slightly above 2 (Table 3 ▸) suggest that there is cooperation between the two active sites of the dimer.

Asp303 O^ɛ2^ is in position to form a hydrogen bond to O2 of the +1 d-xylopyranose moiety (Fig. 2 ▸ *c*). This places Asp303 O^ɛ2^ close to the anomeric C atom and scissile bond of GlcA (we estimate that the distance is ∼2.7 Å; Fig. 2 ▸ *c*), which suggests that Asp303 acts as the catalytic acid, a role that was assigned to Asp206 in *Bo*Agu115A (corresponding to Asp177 in wtsAgu115A; Rogowski *et al.*, 2014 ▸).

In *Bo*Agu115A, it was speculated that Arg299 (Arg328 in *Bo*Agu115A) could play a role in substrate binding, which would have required the enzyme to undergo a conformational change (Rogowski *et al.*, 2014 ▸). The structural data presented for wtsAgu115A do not confirm this claim. An alternative role for Arg299 is in stabilizing the conformation of the putative catalytic acid Asp303, a residue that has also been suggested to be the catalytic acid in *Bo*Agu115A, thus explaining the almost complete loss of activity when this alanine was substituted by an arginine in *Bo*Agu115A (Rogowski *et al.*, 2014 ▸).

Additionally, multiple residues interact with the xylohexaose, which may ensure its optimal orientation, enabling cleavage of the GlcA linkage to O2 of the +1 d-xylopyranose moiety. These include potential hydrogen bonds between Asp221 O^ɛ1^ and O2 or O3 of the +2R d-xylopyranose, between Tyr408 O^η^ and O3 of the +1 d-xylopyranose and between Trp220 O and O3 of the +2R d-xylopyranose, which also stacks against +1NR (Fig. 2 ▸ *b*).

### 3.3. A divalent ion is involved in the formation of the Michaelis–Menten complex and possibly in catalysis

Sites for Na^+^ and Ni^2+^ metal ions have been identified in the crystal structures of *Bo*Agu115A and *Bt*Agu115A, respectively (Aalbers *et al.*, 2015 ▸; Rogowski *et al.*, 2014 ▸). However, the influence of the metal ions on the activity of the enzyme was not investigated.

Three Ca^2+^ ions were modelled in wtsAgu115A D303A in the unbound form. However, two of these are found on the surface of the enzyme (Fig. 1 ▸ *a*) and are likely to originate from the crystallization conditions, which contained 0.2 *M* CaCl_2_. Ca^2+^ site 1 is buried within the structure near the active site (Figs. 1 ▸ *a* and 4 ▸) in a position equivalent to Ni^2+^ in *Bt*Agu115A and Na^+^ in *Bo*Agu115A. Increased activity on an aldouronic acids mixture consisting of (Me)GlcA-xylooligosaccharides of different lengths was observed for EDTA-treated wtsAgu115A in 2 m*M* Mg^2+^ (∼40%) and Mn^2+^ (∼50%), while the presence of 2 m*M* Ca^2+^ resulted in a ∼10% decrease in activity (Supplementary Fig. S4). A kinetic analysis using an aldouronic acids mixture as a substrate surprisingly only resulted in a modest increase in *k*_cat,app_ (14%) in the presence of 2 m*M* Mg^2+^ after EDTA treatment compared with EDTA-treated wtsAgu115A (Table 3 ▸ and Supplementary Fig. S5). Unfortunately, we were unable to obtain structures in complex with (Me)GlcA and aldouronic acids. Superimposition with GlcA from *Bo*Agu115A (PDB entry 4c91) suggests that Asp177, which is involved in the coordination of Ca^2+^ in wtsAgu115A, could interact with (Me)GlcA. Thus, it can be speculated that the smaller Ca^2+^ results in increased distances between the substrate and the residues that interact with it, leading to a slightly decreased activity.

**Figure 4:**
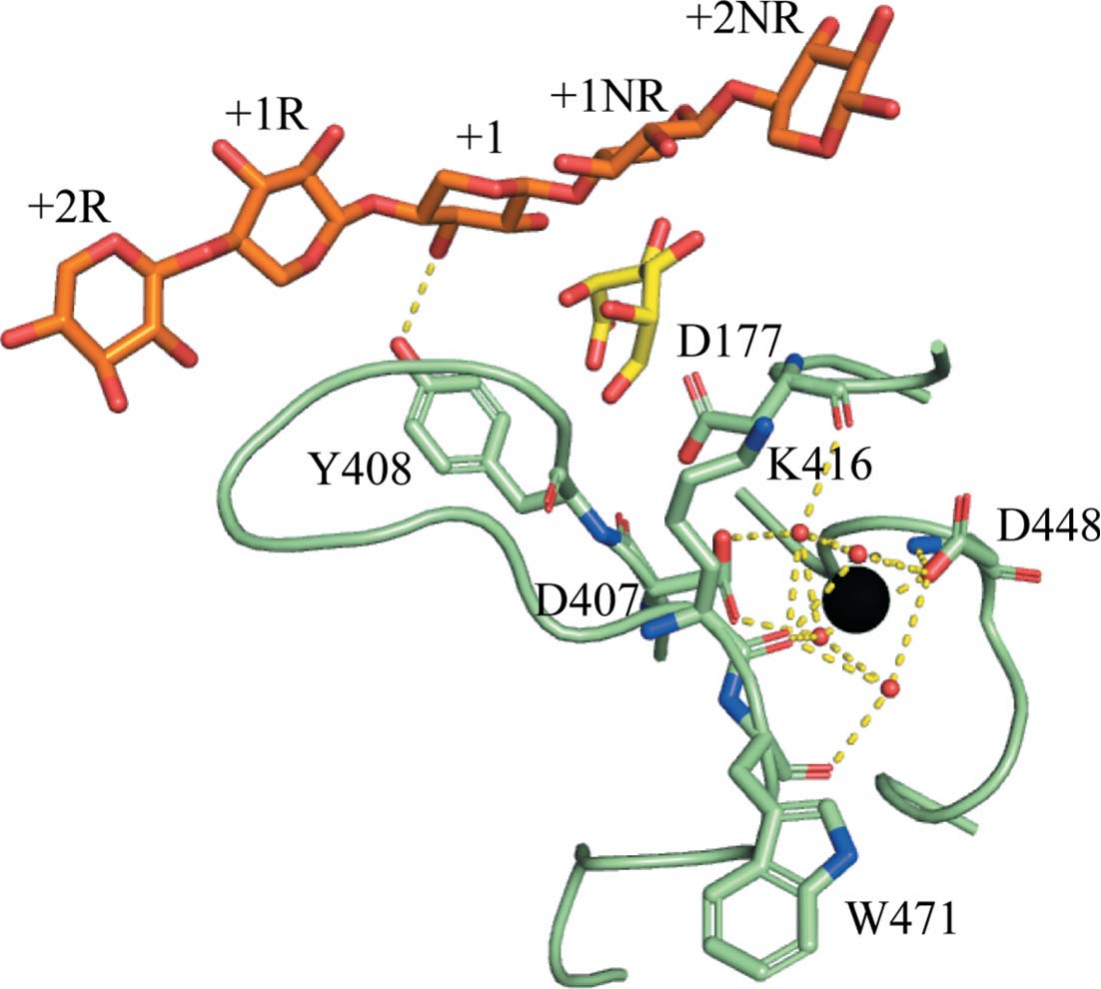
The Ca^2+^-coordinated loops at the active site with residues interacting with either the Ca^2+^ ion (black sphere) or xylohexaose (orange) and glucuronic acid (yellow) from BoAgu115A (PDB entry 4c91). The hydrogen bonds coordinating the waters around the Ca^2+^ ion (black sphere) and interacting with the xylohexaose (orange) are shown by yellow dotted lines (see the text for distances). The subsites are indicated and named according to McKee *et al.* (2012 ▸). d-Xylopyranose moiety 901 corresponds to +2NR, 902 to +1NR, 903 to +1, 904 to +1R and 905 to +2R.

Ca^2+^ site 1 is found to have an octahedral coordination, which in the first coordination sphere is coordinated by Lys416 O and Asp448 O^δ2^ together with four water molecules (water molecules 4, 7, 8 and 16; Fig. 4 ▸). The aforementioned water molecules can form hydrogen bonds to Asp177 O (3.1 Å) and Asp407 O^δ1^ (2.6 Å) or O^δ2^ (2.9 Å) (water molecule 4), Lys416 O (3.0 Å) and Asp448 O^δ2^ (3.4 Å) (water molecule 7), Asp407 O^δ2^ (2.6 Å) and Lys416 O (2.8 Å) (water molecule 8), and Asp448 O^δ2^ (3.5 Å), Trp417 O (2.8 Å) and Lys416 O (3.0 Å) (water molecule 16) in the second coordination sphere (Fig. 4 ▸). The residues involved in Ca^2+^ site 1 are structurally conserved in *Bo*Agu115A, but the most striking difference in *Bt*Agu115A is that Ser395 is located at the position of Asp407 and the water molecules involved in the coordination of the two different metal ions naturally also differ. Ni^2+^ in *Bt*Agu115A is pentahedrally coordinated (Aalbers *et al.*, 2015 ▸). In structurally determined GH115 members consisting of five domains the metal-binding sites are not structurally conserved, which corresponds to the lack of metal ion dependency of the activity for these proteins (Yan *et al.*, 2017 ▸; Wang *et al.*, 2016 ▸).

Two of the three loops (residues 175–179 and 406–421) involved in the coordination of Ca^2+^ site 1 are also involved in substrate binding with Tyr408 O^η^, potentially forming a hydrogen bond to O3 of the +1 d-xylopyranose moiety, which seems to be essential for positioning the scissile bond in the vicinity of the general acid (suggested to be Asp303). Asp177, which was suggested to be the general acid in *Bo*Agu115A (Rogowski *et al.*, 2014 ▸), is involved in coordination of Ca^2+^ site 1 (Fig. 4 ▸) and most likely also interacts with the GlcA moiety. Thus, Ca^2+^ site 1 is directly involved in orientating some of the residues that play a central part in forming the Michaelis–Menten complex.

**Figure 5:**
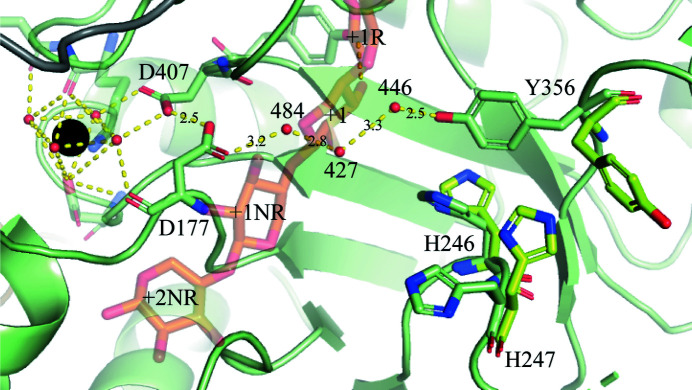
The proton wire that may shuttle the proton needed for catalysis to occur in wtsAgu115A. The Ca^2+^ ion (black sphere), xylohexaose (orange) and hydrogen bonds (yellow dotted lines) with distances indicated in Å are shown for the putative proton wire (see the text for the hydrogen bonds coordinating the waters around the Ca^2+^ ion). Residues shown in lime are from unbound wtsAgu115A D303A and those in pale green are from wtsAgu115A–xylohexaose. The subsites are indicated and named according to McKee *et al.* (2012 ▸). d-Xylopyranose moiety 901 corresponds to +2NR, 902 to +1NR, 903 to +1, 904 to +1R and 905 to +2R.

It was suggested that the residue in *Bo*Agu115A (Asp332) corresponding to Asp303 of wtsAgu115A could be the catalytic base (Rogowski *et al.*, 2014 ▸). However, the structure of wtsAgu115A in complex with xylohexaose suggests, as mentioned, that Asp303 is the catalytic acid, and Asp177 could therefore be the catalytic base. The distance between the scissile bond and Asp177 O^δ1^ is 6.4 Å (Fig. 2 ▸ *c*), which is within the distance of 6–11 Å normally observed in inverting glycoside hydrolases (McCarter & Withers, 1994 ▸). However, from Fig. 4 ▸ it seems more plausible that Asp177 would form hydrogen bonds to GlcA. Further, an alanine mutant of the equivalent to Asp177 in *Bo*Agu115A (Asp206) resulted in a 300-fold reduction in catalytic efficiency (Rogowski *et al.*, 2014 ▸), which would be expected to be greater if Asp177 were the catalytic base. For *Bo*Agu115A it was also mentioned that GH115 xylan-specific α-glucuronidases could employ a Grotthuss-like mechanism (Rogowski *et al.*, 2014 ▸): a proton-transport mechanism in which a proton diffuses through a hydrogen-bond network of water molecules (von Grotthuss, 1806 ▸; Agmon, 1995 ▸). If this were the case, a distant residue could activate the active-site water nucleophile, which was also suggested to be the case for cellulases in glycoside hydrolase family 6 (Brás *et al.*, 2011 ▸; Mayes *et al.*, 2016 ▸). The wtsAgu115A–xylohexaose structure revealed a proton wire leading from Ca^2+^ site 1 to Tyr403 through the active-site cavity via water molecules w427, w446 and w484 (Fig. 5 ▸; see Supplementary Fig. S6 for an omit map) and likely beyond Ca^2+^ site 1. Thus, the proton required for catalysis could be delivered through this proton wire. Interestingly, upon formation of the Michaelis–Menten-like complex, Tyr403 moved nearly 170° and the nearby histidine residues His246 and His247 also underwent conformational changes (Fig. 5 ▸), indicating the importance of Tyr403 in particular in catalysis. This is supported by the 10^−3^ loss in activity observed for an alanine mutant in *Bo*Agu115A (Rogowski *et al.*, 2014 ▸). In both *Bo*Agu115A and *Bt*Agu115A the corresponding tyrosine is in a conformation similar to that observed in wtsAgu115A in complex with xylohexaose; thus, the movement is not necessarily dependent on the substrate interaction. Further studies are needed to determine how the proton travels via the proton wire and thus determine the exact role of Tyr403 in catalysis. It would be interesting to see how Mg^2+^ and Mn^2+^ affect the divalent metal-binding site and thus substrate binding and the proton wire.

### 3.4. GH115 phylogeny and metal-binding sites

A phylogenetic tree was constructed to allow an analysis of the grouping of GH115 members in relation to their metal dependence. The xylan α-1,2-glucuronidases are grouped together in the lower clade (Fig. 3 ▸), while *Bt*GH115A from *B. thetaiotaomicron* shown to hydrolyse (Me)GlcA on arab­ino­galactans is found in the left clade (Fig. 3 ▸). wtsAgu115A is found in a clade distant from other xylan α-1,2-glucuronidases together with *Bo*Agu115A. *Bo*Agu115A and wtsAgu115A segregate on neighbouring clades (Fig. 3 ▸). Most of the interactions with Ca^2+^ site 1 in wtsAgu115A are with backbone atoms (Fig. 4 ▸). However, for Asp407 it is the side-chain atoms that coordinate the water molecules around Ca^2+^ site 1, which together with the fact that in the Ni^+^-containing *Bt*Agu115A Asp407 is replaced by Ser395, suggests that the presence of aspartic acid in this position could be an indication of a divalent metal-binding site. This is the case for about two thirds of the CAZy GH115 members (Fig. 3 ▸), but unfortunately the metal dependence was not tested for *Bo*Agu115A (Rogowski *et al.*, 2014 ▸) or *Sp*Agu115A from *Streptomyces pristinaespiralis* (Fujimoto *et al.*, 2011 ▸), which are suggested to have a divalent metal-binding site (Fig. 3 ▸).

Although *Bo*Agu115A and wtsAgu115A are found in neighbouring clades, an exciting difference between the two clades involves the loop harbouring the suggested general acid Asp303. The 15 sequences constituting the wtsAgu115A clade all share a unique 12-amino-acid insert (K-E-G-E-D-D/H-L/K-Y/F-V-P/S-R/S-D) in the loop mentioned above (Fig. 3 ▸). A single sequence (GenBank Accession No. AVM57875.1) from *Prevotella heparinolyticus* that segregates from the wtsAgu115A and *Bo*Agu115A clades (Fig. 3 ▸) contains an insert very similar to that found for the wtsAgu115A clade members (E-E-G-K-D-H-E-Y-V-A-R-Y; Fig. 3 ▸). Yan *et al.* (2021 ▸) also observed variance in both the amino-acid composition and the length of the aforementioned loop and suggested that it determines the substrate specificity of GH115 members and indicates that GH115 members with longer flexible loops can accommodate highly substituted xylans. Further studies involving such substrates are needed to confirm this hypothesis.

## 4. Biochemical characterization

The pH optimum of wtsAgu115A using aldouronic acids as a substrate was pH 6 (Supplementary Fig. S7*a* ). After incubation for five days, wtsAgu115A retained activity in the pH range 6–11, while no activity was detected in the pH range 2–5 after five days of incubation at room temperature (Supplementary Fig. S7*b* ).

The temperature optimum of wtsAgu115A using aldouronic acids was determined to be close to 40°C (Supplementary Fig. S7*c* ). This is not surprising as wtsAgu115A was identified in the metagenome from a mesophilic anaerobic digester (Wilkens *et al.*, 2017 ▸). 90 ± 4% of the activity of wtsAgu115A was retained while incubating at 37°C for 23.3 h; the activity then decreased to 67 ± 4% after 78.1 h (Supplementary Fig. S7*d* ). wtsAgu115A retained 4 ± 0.7% of its activity after 5 min incubation at 50°C, while no activity was detected after 10 min incubation at 50°C.

## Supplementary Material

PDB reference: GH115 α-1,2-glucuronidase, 7pxq

PDB reference: complex with xylopentaose, 7pug

Supplementary File S1. Alignment file for glycoside hydrolase family 115. DOI: 10.1107/S2059798322003527/lp5057sup1.txt

Supplementary Figures. DOI: 10.1107/S2059798322003527/lp5057sup2.pdf

## Figures and Tables

**Table 1:** Data collection and processing Values in parentheses are for the outer shell.

Ligand	None	Xylohexaose
PDB entry	7pxq	7pug
Data collection		
Beamline	BioMAX	P14
Wavelength (Å)	0.9762	0.9762
Resolution (Å)	50.47–2.30 (2.38–2.30)	65.26–2.66 (2.76–2.66)
Space group	*P*6_1_22	*P*6_1_22
*a*, *b*, *c* (Å)	148.2, 148.2, 274.5	148.6, 148.6, 272.8
α, β, γ (°)	90, 90, 120	90, 90, 120
Total No. of reflections	1597035 (150697)	871723 (87660)
No. of unique reflections	79371 (7781)	51774 (5062)
*R*_merge_	0.10 (3.09)	0.11 (3.32)
*R*_meas_	0.11 (3.18)	0.12 (3.42)
*R*_p.i.m._	0.02 (0.71)	0.03 (0.82)
CC_1/2_	1.00 (0.46)	1.00 (0.37)
〈*I*/σ(*I*)〉	17.30 (0.86)	17.87 (0.80)
Completeness (%)	99.93 (99.63)	99.94 (99.88)
Multiplicity	20.1 (19.4)	16.8 (17.3)
Refinement		
Reflections used	79346 (7768)	51755 (5056)
Reflections used for *R*_free_	4033 (409)	2574 (256)
*R*_work_	0.19 (0.30)	0.17 (0.49)
*R*_free_	0.22 (0.31)	0.21 (0.50)
CC_work_	0.96 (0.73)	0.95 (0.68)
CC_free_	0.95 (0.72)	0.94 (0.74)
No. of refined non-H atoms		
Total	6982	7122
Protein	6708	6762
Ligand	3	104
Solvent	271	298
Average *B* factors (Å^2^)		
Overall	83.0	99.1
Protein	83.5	99.4
Ligand	83.3	131.1
Solvent	71.2	84.8
Wilson *B* factor (Å^2^)	67.0	88.2
R.m.s.d.		
Bond lengths (Å)	0.008	0.008
Angles (°)	0.91	0.92
Ramachandran plot (%)		
Favoured	95.96	94.06
Allowed	3.92	5.58
Outliers	0.12	0.36
Rotamer outliers	4.99	6.33
No. of TLS groups	10	3

**Table 2:** Specific activities of wtsAguGH115A n.d., not detected. Relative values are in parentheses. All experiments were performed in triplicate.

Substrate	Specific activity (U mg^−1^)
Aldouronic acids	0.063 ± 0.009 (1.00)
Beechwood xylan	0.038 ± 0.001 (0.60)
Birchwood xylan	0.017 ± 0.002 (0.27)
Sugar beet L-arabinan	n.d.
Linear L-arabinan	n.d.
Wheat arabinoxylan (insoluble)	n.d.
Wheat arabinoxylan (low viscosity)	n.d.
Rye arabinoxylan	n.d.
Corn cob xylan	n.d.
Oat spelt xylan	n.d.
Potato pectic fibre rhamnogalacturonan I	n.d.
Soy bean pectic fibre rhamnogalacturonan	n.d.
Acacia tree gum arabic	n.d.
Xanthan gum	n.d.
Larch wood arabinogalactan	n.d.
Potato galactan	n.d.
Tamarind seed xyloglucan	n.d.

**Table 3:** Apparent kinetic parameters of wtsAgu115A towards an aldouronic acids mixture in the presence and absence of MgCl_2_

	*V*_max,app_ (g MeGlcA l^−1^ min^−1^)	*K*_m,app_ (g l^−1^)	*n*	*k*_cat,app_ (s^−1^)
Without MgCl_2_	0.08 ± 0.01	0.96 ± 0.19	2.11	1.30
With MgCl_2_ (2 m*M*)	0.09 ± 0.01	1.05 ± 0.18	2.23	1.48
