# Supplementary material for: A GH115 α-glucuronidase structure reveals dimerization-mediated substrate binding and a proton wire potentially important for catalysis
Source: Acta Crystallogr D Struct Biol. 2022 Apr 20;78(Pt 5):658–68. doi: 10.1107/S2059798322003527 (PMC9063842; doi:10.1107/S2059798322003527)
Supplement: Supplementary file 2 [file d-78-00658-sup2.pdf]

Acta Cryst D

STRUCTURAL BIOLOGY

Volume 78 (2022)

Supporting information for article:

A GH115 α-glucuronidase structure reveals dimerization-mediated substrate binding and a proton wire potentially important for catalysis

Casper Wilkens, Marlene Vuillemin, Bo Pilgaard, Igor Polikarpov and Jens Preben Morth

CCATGGATAACAAGGGAATAACATACGAAGAACTAAACCCCGAAAGATTCACTCTATTAGAGAAAGGT
ACCCCGACCAACATCCTGATTGACGAAAACGAGGATCAGGGCGTGATGATTGCGGCGACCAACCTGAG
CGAAGACTTCGGTCGTGTTAGCGGCACCAACGCGCCGCTGATCTTTCTGCCGGATAACGAACGTCTGA
TCATTGTGGGTACCCTGGAGAGCCGTTATATTAAGGAACTGACCGAGAACCGTAAGATCAAAGGTGAC
GAACTGAAGGGCAAAAACGAGAAATACCTGATGACCGTGGTTGATAACCCGCTGCCGGGTGTGAAAGA
GGCGCTGATCATTGCGGGCAGCGACAAACGTGGTGCGATCTACGGCATTTATGAACTGAGCGAGCAGA
TTGGTGTTAGCCCGTGGTATGACTGGGCGGATGTGCCGGTTAAGCCGCAGCAAAACCTGAGCATTGAA
CGTGGTAGCTACACCGCGGATGAGCCGGCGGTTACCTATCGTGGCATCTTCCTGAACGATGAGGCGCC
GGCGCTGACCAGCTGGGTGGAGAACACCTACGGTACCAAATATGGCGACCACCGTTTCTATAGCCGTG
TTTTTGAGCTGATCCTGCGTCTGCGTGGTAACTTCCTGTGGCCGGCGATGTGGGACTGGAGCTTTTAC
GGCGACGATCCGCTGAACAGCAAGACCGCGGATACGATGGGTATCATTATGGGTACCAGCCACCATGA
ACCGATGGCGCGTAACCACCAGGAGTGGGCGCGTAACCGTGACAAATACGGTGTGTGGGATTATACCA
GCAACCAGGAAGTTATTGACCAATTCTTTCGTGAAGGTATCGAGCGTGTGAAGGATACCGACGATCTG
ATCACCATTGGCATGCGTGGTGACGGCGATACCCCGATGGGTGTGAAAGAAGGCGAGGACCACCTGTT
TGTTCCGAGCGACGAAGATAACATGCGTCTGCTGGAGCGTATCATTAAGAACCAACGTGAGATCATTG
GTGATGTTACCGGTGAAAGCCCGGAGAAGACCCCGCAGGTTTGGGCGATTTACAAAGAAGTTCAACGT
TATTTCGACCTGGGTCTGCGTCCGCCGGAGGATGTGATCATTCTGCTGAGCGACGATAACTGGGGCAA
CGTTCGTCGTCTGCCGACCGAGGAAGAGCGTGACCACCCGGGTGGCTGGGGCATGTACTATCACTTTG
ATTATGTTGGTGCGCCGCGTAGCAGCAAGTGGCTGAACATCAGCCCGATTCAGAACATCTGGGAACAG
ATGCAACTGACCTACGACTATGGTGTTGATGAGCTGTGGGTGGCGAACGTTGGCGACCTGAAACCGAT
GGAATACCCGATCACCCTGTTCCTGGACATGGCGTGGGATCCGACCCGTTTTAACGCGGAGAACCTGC
TGGATCACACCCGTAGCTTCGCGGCGCAGCAATTTGGTGAAGACCAGGCGGATGAGGCGGCGCGTATC
ATTAACCTGTACAGCAAGTATAACGGTCGTGTGACCCCGGAAATGCTGGACCGTAACACCTACAACCT
GGAAAGCGGCGAGTGGAAGAAAGTTAGCGATGAGTACATTAAGCTGGAAGCGGAGGCGCTGCGTCAAT
ATCTGACCCTGGAACCGGAGCAGCGTGACGCGTACAAACAACTGATCCTGTATCCGGTGCAGGCGATG
GCGAACCTGTACGAAATGTACTATAGCCAAGCGATGAACCACAAACTGTACCGTGAGAACAACCCGAT
GGCGAACTATTGGGCGGACCGTGTGGAAGAGACCTTCAACCGTGACGCGGAGCTGAGCCACGATTATA
ACAAGGTTATGGCGAACGGTAAATGGGATGGCATGATGACCCAGAAGAAAATTGGTTACCGTAGCTGG
AACGACAACTTCCCGGCGGATACCCTGCCGCAAATTTTTCGTATCGAAAACCCGGAAGAGGCGACCGG
TGGCTACGTGTTCACCGCGCGTGACGGCGTGGTTGTGATCGAAGCGGAGCACTATTTTGAGGCGAAGG
ATGCGGAAGAGGCGAAATGGACCGTTATTCCGTACATGGGTCGTACCCTGAGCAGCATCGCGCTGATG
CCGTACACCAAAGAAGTGGAGGGCGCGAGCCTGAGCTATCGTATGCAGATCCCGGACGAAGTTAGCGA
GGTGAAGGTTCACGTTGTGGTTAAAAGCACCCTGCCGTTCCACGATCCGAAGGGTCACGAATACCGTG
TGGGCTTCGAAGGTGGCAGCAAAGAGATTGTTAACTTTAACTGGAACCTGAACGAAGAGCCGGAGAAC
ATCTACAGCGTGTTCTATCCGACCGTTGCGAGCCGTGTGGTTAAGAAAGACGTGACCCTGGACCTGCA
CGATACCGACGATGGTTTTTATACCCTGACCCTGGAACCGCTGGACCCGGGTATTGTTTTCCAAAAGA
TCGTGGTTGATTTTGGTGGCTACGAAGAGAGCCGTCTGTTTATGGAAGAGAGCCCGAACAAACGTATC
GAGAGCCGTCTCGAG

Figure S1 Escherichia coli codon optimised gene encoding for wtsAgu115A.

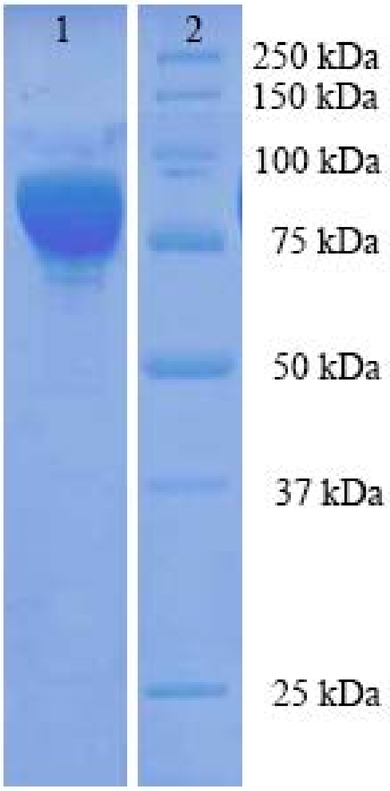

Figure S2 SDS-PAGE of wtsAgu115A. Lane 1 shows wtsAgu115A and lane 2 the marker with sizes indicated in kDa.

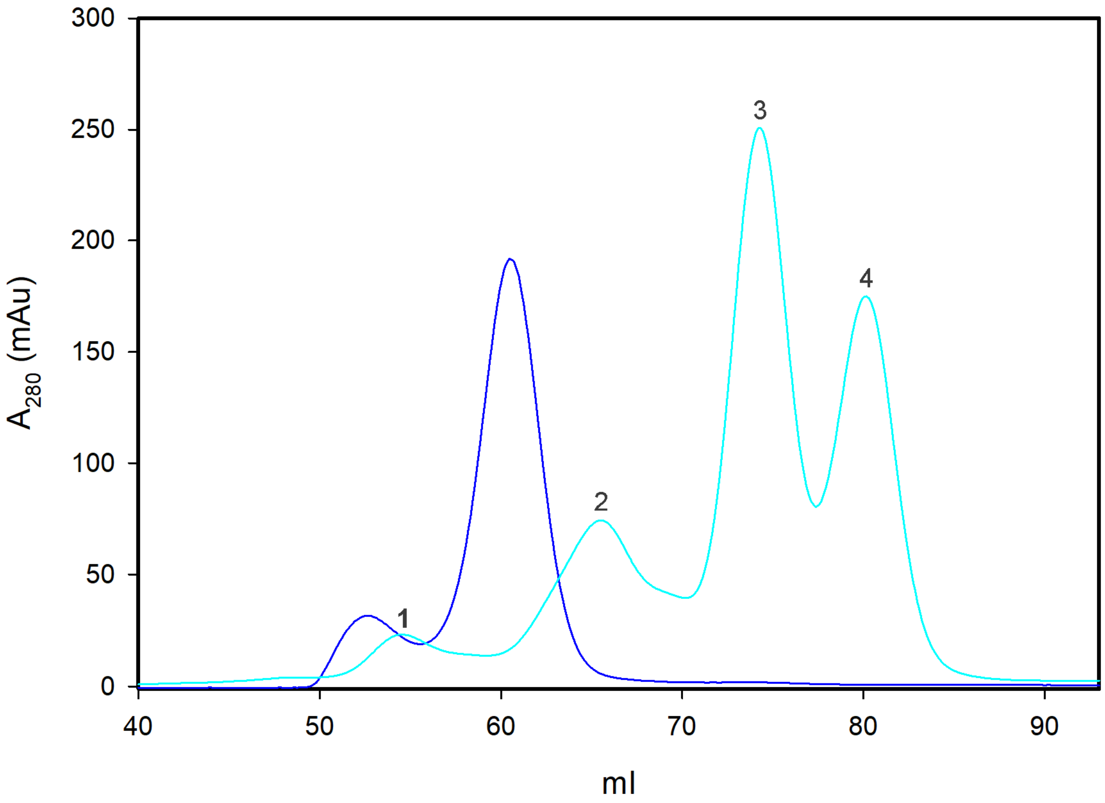

Figure S3 Analytical size exclusion chromatography wtsAgi115A (blue) compared to ferritin (1), aldolase (2), conalbomin (3) and ovalbumin (4) (cyan).

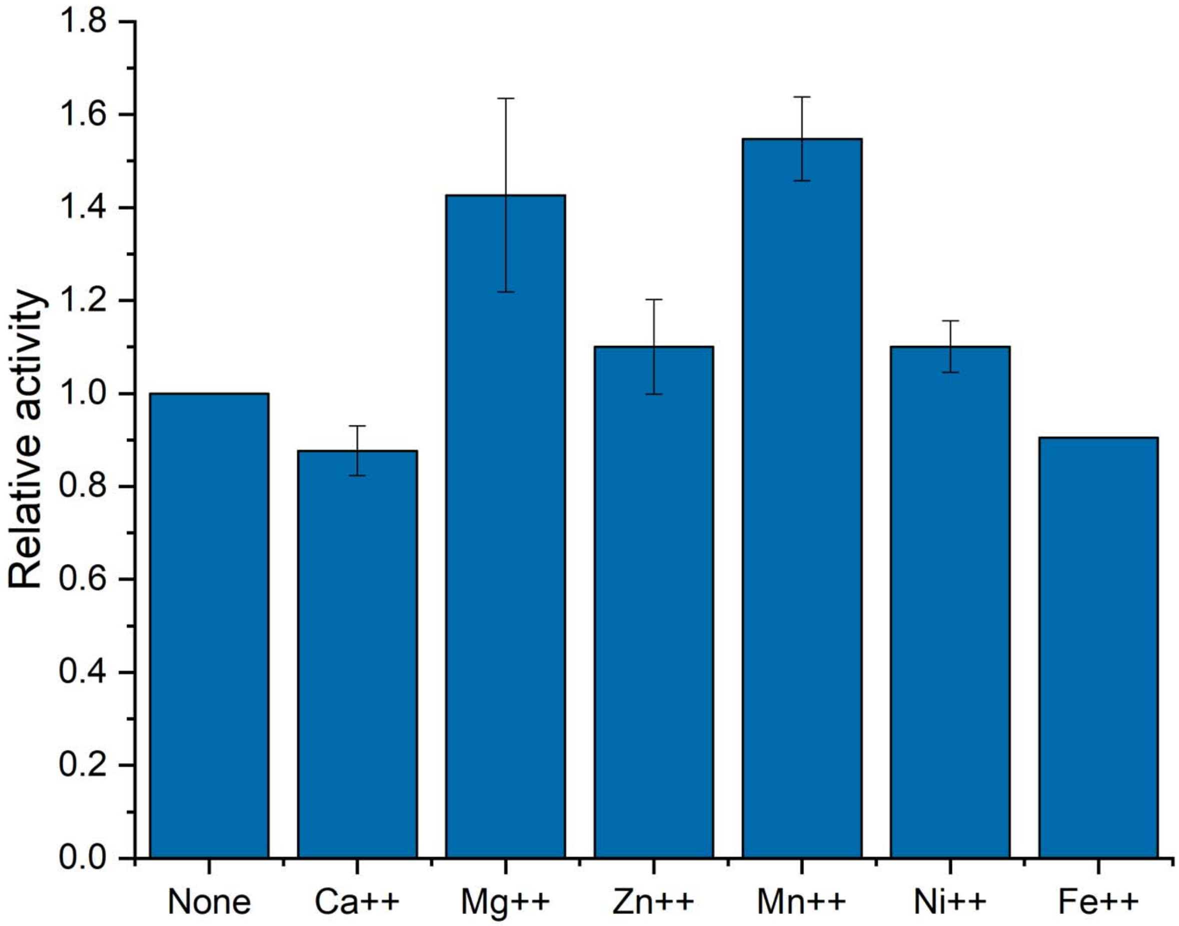

Figure S4 Effects of divalent metal-ions on wtsAgu115A activity on aldouronic acids.

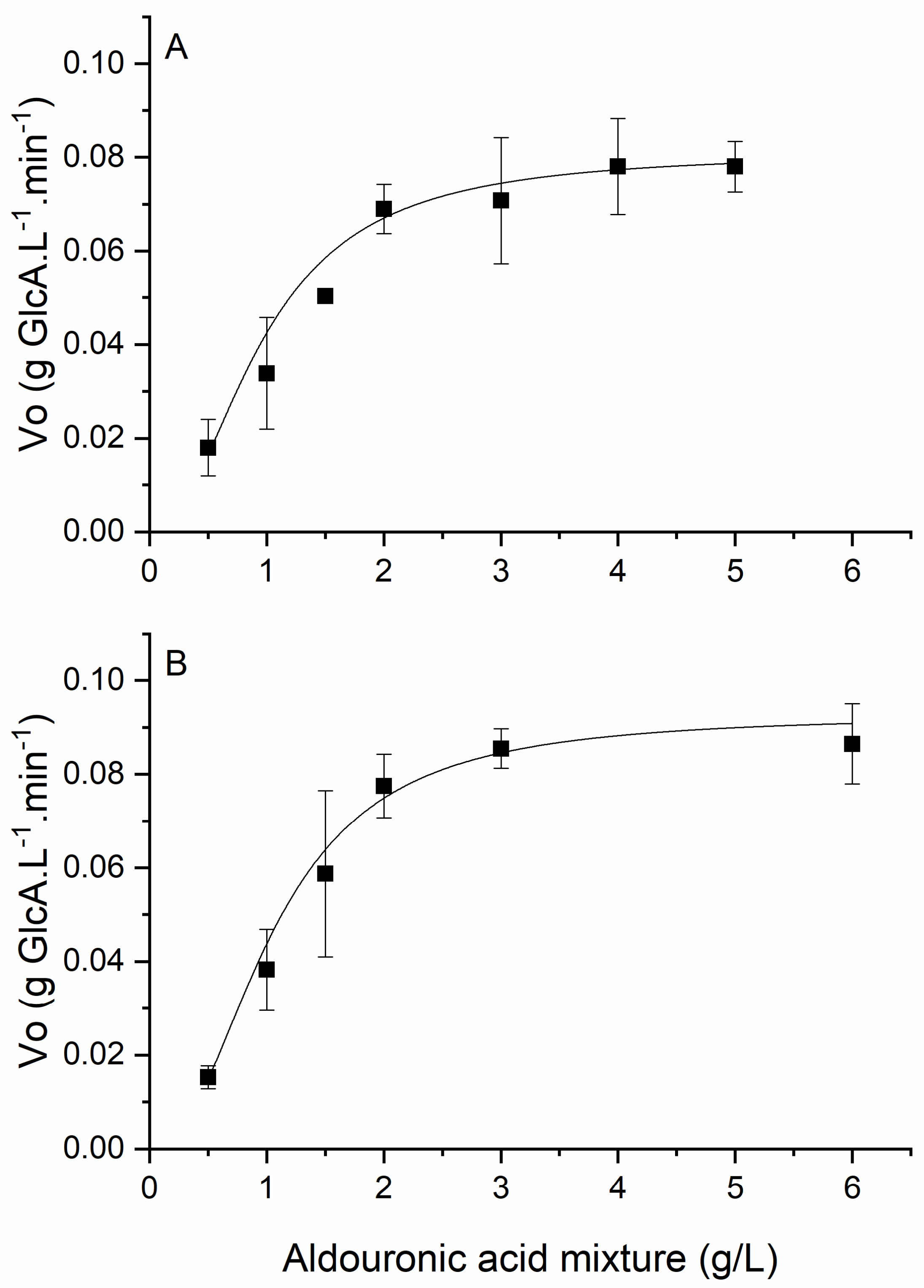

Figure S5 Substrate hydrolysis curves for wtsAgu115A of A) aldouronic acids and B) aldouronic acids in the presences of 2 mM MgCl.

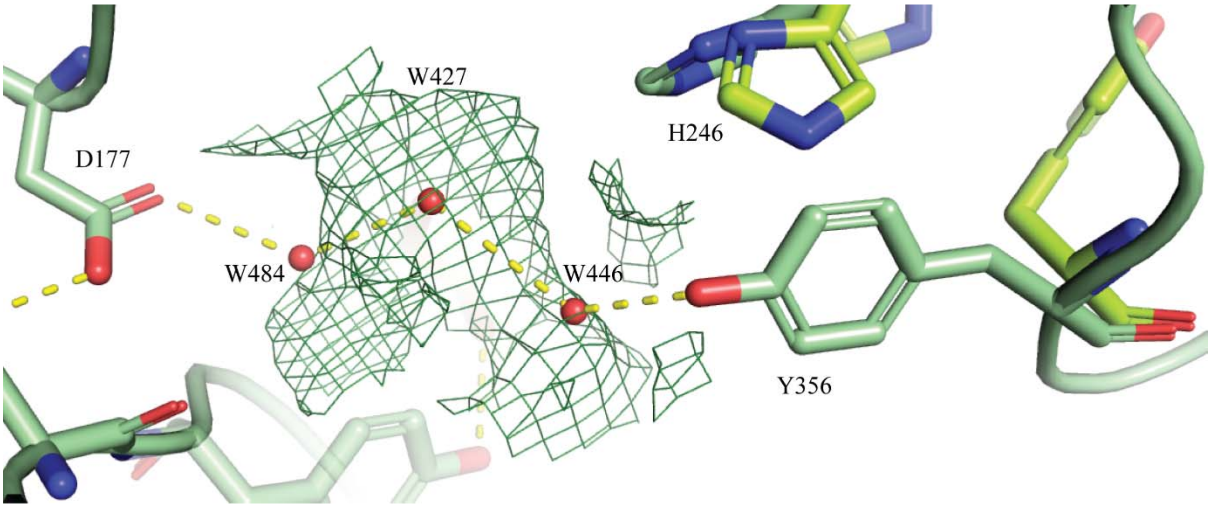

Figure S6 Omitmap (contoured to 3.0σ in green mesh with a cutoff at 3.0 Å for C) of wtsAgu115A water molecules 427, 446 and 484 (red) with xylohexaose (orange) and hydrogen bonds (yellow dotted lines) , and residues shown in lime are from wtsAgu115A-D303A-unbound and in pale green are from wtsAgu115A-xylohexaose.

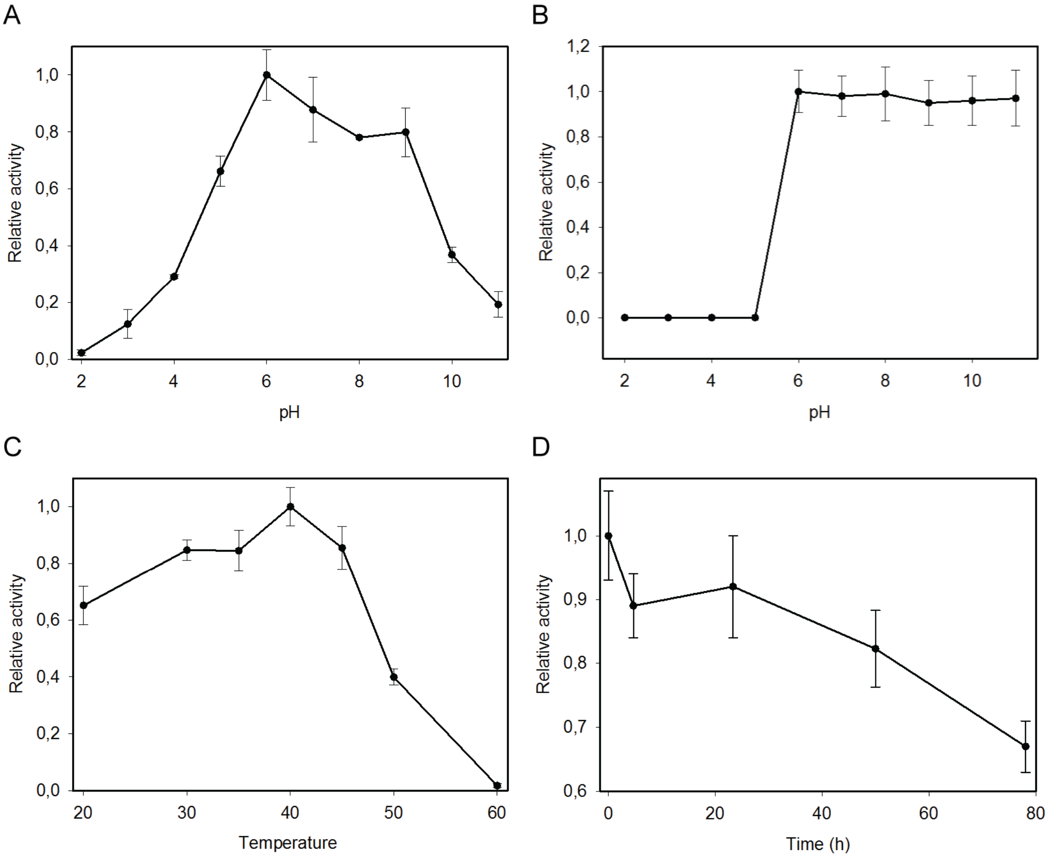

Figure S7 wtsAgu115A A) pH optimum, B) pH stability, C) temperature optimum and D) stability at 37 ºC.
